# The Canada Journal of Dental Science

**Published:** 1868-06

**Authors:** 

**Affiliations:** Montreal; Toronto


### THE CANADA JOURNAL OF DENTAL SCIENCE.

Edited by W. G. Beers, Montreal, and J. S. Scott, M. D., Toronto.

This is a well gotten up Journal of 32 pages, published monthly. We hail its advent as a good omen for the profession in Canada, and indeed wherever else the Journal may circulate. Its pages are well filled with interesting and instructive matter. The reputation of its editors is a guarantee of what the Journal will be. Long may it live, and great may be its influence for good. Price, $3 00 per annum. T.

